# Assessing the psychometric properties of the Symptom Checklist Five-Items (SCL-5) instrument in a clinical setting for treating intimate partner violence

**DOI:** 10.1186/s12888-026-08522-y

**Published:** 2026-08-14

**Authors:** Merete Berg Nesset, Tom Palmstierna, Richard Whittington, Mariela Loreto Lara-Cabrera

**Affiliations:** 1 St. Olav’s Hospital, Clinic of Mental Healthcare – General, Rehabilitation and Security, Department of Security, Prison and Forensic Psychiatry, Centre for Research and Education in Security, Prison and Forensic Psychiatry, Trondheim University Hospital, Trondheim, Norway; 2 Department of Mental Health, Faculty of Medicine and Health Sciences, Norwegian University of Science and Technology (NTNU), Trondheim, Norway; 3 Department of Clinical Neuroscience, Centre for Psychiatric Research, Karolinska Institutet, Stockholm, Sweden; 4 School of Humanities and Social Sciences, University of Chester, Chester, UK; 5 Nidelv Community Mental Health Centre, St. Olav’s Hospital, Trondheim University Hospital, Clinic of Mental Healthcare, Trondheim, Norway

**Keywords:** Anxiety, Assessment, Depression, Domestic violence, Intimate partner violence, Group therapy, Outcome assessment (health care), Psychometrics, Reliability, Validity

## Abstract

**Background:**

Research suggests that the Symptom Checklist Five Items (SCL-5) instrument is a reliable and valid scale for assessing mental health in a range of populations. Yet, to date, the SCL-5 has not been validated in a population of perpetrators of intimate partner violence (IPV) and their partners. Hence, the current study aimed to examine the psychometric properties of the SCL-5 within a clinical IPV context characterized by elevated mental health symptoms.

**Methods:**

This validation study is part of a randomised controlled trial evaluating a group intervention for men voluntarily seeking treatment for IPV in Norway. The study was conducted in accordance with the guidelines Consensus-based standards for the selection of health measurement instruments (COSMIN). We evaluated floor and ceiling effects, internal consistency and estimated the area under the curve to determine optimal diagnostic sensitivity and specificity. Additionally, we explored scale responsiveness using data from a subsample of participants.

**Results:**

The highest percentage of respondents with the lowest possible score (value 1) across the 5 items was 8.7%, indicating no floor effect assuming the 15% criterion. The highest percentage with the highest possible score (value 4) across the 5 items was 1.9%, indicating no ceiling effect either. Cronbach’s alpha was 0.86 for the total sample (*N* = 161), 0.83 for perpetrators only (*n* = 118) and 0.89 for partners only (*n* = 44). The area under the curve was 94.6% (95% CI 0.913-0.978) for the total sample, 93.5% (95% CI 0.893-0.977) for the perpetrators and 96.9% (95% CI 0.922–1.017) for the victims. Regarding responsiveness, we set a criterion of a statistically significant reduction in mean scores over time as a test of the adequate responsiveness of the SCL-5 from the baseline to the one-year follow-up point. As predicted, it fell from 1.98 to 1.74 and the mean change was significant (*t* −4.021, CI −0.360 to −0.121; *p* < 0.001). The mean total SCL-5 score decreased in 54% of cases *(n* = 43).

**Conclusions:**

The results provide evidence that the SCL-5 has adequate psychometric properties in a Norwegian sample of perpetrators and victims of IPV.

**Clinical trial number:**

Not applicable.

## Introduction

Over the last several decades, researchers and clinicians have developed specific short, self-report measures like the Hopkins Symptom Checklist-25 (HSCL-25) and its briefer derivative, the Symptom Checklist Five-Items (SCL-5), to measure symptoms of anxiety and depression. The HSCL-25 [1, 2] is widely used to identify individuals with high levels of emotional distress and it has been validated in several languages, including Spanish [3], Arabic [4], Urdu [5], Swedish [6, 7], Norwegian [8], Indochinese [9], German [10], and French [11]. However, there is a trade-off between brevity and accuracy regarding how short any checklist should be. Shorter checklists may appear more desirable to use amongst people with mental health and cognitive processing challenges like, for instance, violent individuals with comorbid depression and anxiety [8, 12]. From both a research and clinical perspective brief instruments may reduce respondent burden and improve completion rates [13] but may compromise the psychometric qualities of the longer version.

Loss to follow-up is a major threat to the validity of randomised controlled trials and clinical studies relying on self-reported outcome measures [14]. Therefore, having a brief and easy-to-use self-report questionnaire can be useful if it has robust psychometric properties. Despite the practical advantages of the SCL-5, to the best of our knowledge there have been no previous estimates of its properties amongst perpetrators as a specific clinical group. Progress in research on IPV has until now been hampered by methodological challenges, like, for instance, the use of non-validated instruments when measuring different aspects of mental health issues, which makes it difficult to compare results between studies [15]. Therefore, taken together, these issues suggest the need for validation studies of brief checklists which are easy to use.

### Previous validation research on the symptom check list

There are several comparative validation studies investigating the psychometric properties of the SCL-5. Five different studies examined these properties in a sample of the German [16], Spanish [17], and Norwegian general populations [8, 18, 19], respectively, and all five studies found that the SCL-5 has good psychometric properties in these populations. The SCL-5’s internal consistency has previously been reported to be between 0.77 and 0.87 [8, 17]. However, it has not been tested beyond the general population (e.g. in clinical samples) in Norway.

### Intimate partner violence and mental health

Intimate partner violence (IPV) is a major challenge from a public health perspective [20].

A recent systematic review of mental health outcomes associated with IPV has suggested increased odds for depression, post-traumatic stress disorder, and suicidality amongst female victims [21]. The global lifetime prevalence of IPV exposure for ever-married women over 15 years has been estimated as 26%, with wide country-level variations [20]. IPV comprises physical, sexual, psychological and social violence including verbal threats, coercive tactics, and controlling of the family economy and social relationships by the partner [22, 23]. Both men and women can be subjected to IPV, although research has shown that the consequences of violence are more serious amongst women [20]. Experiencing IPV is related to a long-term negative impact on the victims’ mental and physical well-being [21, 24].

### Anxiety, depression and anger as factors in intimate partner violence

Whilst most people with mental health challenges are not violent, research has indicated that anxiety and depression are associated with the perpetration of IPV [12, 21, 25]. Furthermore, in specialized clinical settings, anxiety and depressive symptoms are linked to difficulties in emotion regulation, irritability and maladaptive coping strategies, which in some individuals may contribute to conflict escalation within intimate relationships [12]. Research examining men voluntarily attending therapy for IPV behaviour has found that approximately 43% of the sample had high levels of depression and/or anxiety [12]. Mental health care professionals should pay particular attention to anxiety and depression comorbidities when presented with patients with anger problems. Moreover, mental health practice must evolve by screening and measuring the full range of mental health challenges to help those involved in IPV [8, 12, 26, 27].

Psychological interventions for people who perpetrate IPV are typically based on a cognitive-behavioural model targeting angry feelings which predispose the person to act aggressively [28, 29]. The intervention used in this study fitted with this approach and the primary outcome of the overall evaluation was anger and aggression. However, anxiety and depression are also important features of the IPV profile for both perpetrators [30] and their victims [31] so these populations are suitable for the task of testing the SCL-5. Domestic violence perpetrators who score high on the Conflict Tactics Scale, for example, also show high levels of PTSD, generalised anxiety disorder, panic disorder and depression [32]. In theoretical terms there is a consensus that anxiety, depression and anger share common physiological pathways which only generate divergent emotions as a result of the psychological process of cognitive appraisal [33]. There is also substantial diagnostic comorbidity within psychiatric taxonomies with irritability listed in DSM5 as a symptom of generalized anxiety disorder and depression and anxiety associated with intermittent explosive disorder [34]. The ‘Brøsetmodellen’ intervention used here is focused on anger in IPV perpetrators but includes several psycho-educative sessions where core CBT concepts such as negative thoughts, thinking errors and coping strategies are discussed [35]. These concepts are generic components of emotion regulation, and it is likely that fostering them in therapy will enable improvements in managing feelings of anxiety and depression alongside anger. It is reasonable to suppose, therefore, that an anger management intervention like this may change the intensity of other emotions, including anxiety and depression, to a degree that will be picked up by the SCL-5.

### The current study

The current study aimed at examining the psychometric properties of the SCL-5 amongst a Norwegian sample of IPV perpetrators and their intimate partners. The primary aim of the study was to examine the psychometric properties of the SCL-5 within a clinical IPV context characterized by elevated mental health symptoms. The specific objectives were to examine distributional properties, internal consistency, reliability and responsiveness to change at 1-year follow-up. By assessing these properties, we sought to determine whether the SCL-5 is a reliable and clinically useful brief screening instrument for anxiety and depression symptoms in IPV-related treatment settings. The focus was on scale performance rather than group comparisons.

The study was conducted in accordance with the COSMIN guidelines [36–38] which set out best practice in evaluating health-related outcome measures by using the following pre-defined hypotheses for judging an adequate outcome measure instrument. In our first hypothesis, we predicted no floor or ceiling effects in the distribution of SCL-5 responses. In our second hypothesis, based on another Norwegian study, we proposed that the SCL-5 would have a Cronbach’s alpha of 0.7 or higher [8]. In the third hypothesis we predicted that the SCL-5 would detect clinical cases as identified by the full HSCL-25 scale in the total sample (perpetrators and partners combined), as well as in the perpetrator and victim sample separately. This was examined by calculating the area under the curve (AUC) of receiver operating characteristics (ROC) to establish the optimal trade-off between sensitivity and specificity in identifying caseness. In our fourth hypothesis, we predicted that the SCL-5 would be able to detect a reduction in anxiety and depression from the baseline to the one-year follow-up point.

## Methods

### Study design and participants

As part of a programme to support mental health professionals tasked with screening and treating individuals perpetrating IPV, we extracted self-reported data from a previously published clinical trial involving male IPV perpetrators and their intimate partners [12].

All the men were undergoing treatment voluntarily. The perpetrators and their intimate partners had completed the SCL-5 at baseline and at one-year post-treatment follow-up.

The sample was recruited from a study of the effectiveness of group therapy (GT) on recurrent violent behaviour towards intimate partners. Participation was based on written consent provided by the participants after receiving verbal and written information about the study. The enrolment period ran from 2012 to 2018. Inclusion criteria were that the perpetrators and their partners were 18 years of age or older, spoke Norwegian fluently and (perpetrators only) were willing to attend the GT. Fluency was assessed during the intake process by clinicians conducting structured screening interviews. Participants were required to demonstrate sufficient oral and written Norwegian proficiency to complete questionnaires without interpreter assistance. Exclusion criteria were perpetration of violence towards non-partners only, drug use or current uncontrolled psychotic symptoms.

To examine the psychometric properties of the SCL-5 we used data from all participants (perpetrators and partners) who had completed the SCL-5 at the baseline. To examine the responsiveness issue, we used data from 80 of these participants who completed the SCL-5 both before and after the intervention (baseline and one-year follow-up).

### Intervention for perpetrators

The perpetrators received a combination of individual sessions and group-based therapy. They were randomly allocated to receive either cognitive behavioural group therapy (CBGT) or mindfulness-based stress reduction therapy (MBSR). The CBGT intervention consisted of a manualised 15-week group programme (for a total of 30 hours), with two additional individual sessions prior to group attendance. Through structured Socratic dialogue, therapists and participants examined prior violent incidents to identify how maladaptive beliefs triggered situation-specific negative thoughts, emotional dysregulation, and misinterpretations of the partner’s intentions. Participants completed weekly homework to practice non-violent strategies. The MBSR treatment consisted of eight group sessions (for a total of 16 hours) of mindfulness-based stress reduction group therapy plus two additional individual sessions each occurring before and after the group programme [39]. Both therapies aimed at enhancing emotion regulation and changing ineffective communication patterns by addressing negative thoughts and feelings in certain situations [12]. In addition, the cognitive behaviour therapy addressed anger and aggressive behaviour by targeting behavioural change strategies and modifying core beliefs [35, 40].

### Intervention for intimate partners

As the intimate partners were victims of IPV, it was essential for clinical services to provide support designed to maximize their well-being. Therefore, the outpatient service offered psychoeducation about the treatment process and encouraged the intimate partners to call a victim support centre at any time if they felt unsafe. They were also told to call the police if they feared for their safety. The therapists had a duty by law to notify the children’s welfare services or the police if they were concerned about the safety or well-being of the victims.

### Measurements

Information about the participants’ age, mother tongue, education level, family situation, and employment status was collected at baseline.

#### Hopkins symptom checklist- 25 and SCL-5

The HSCL-25 [1, 2] is a screening questionnaire designed to identify symptoms of anxiety and depression as experienced over the preceding two weeks by adults. The HSCL-25 has 10 items relating to anxiety and 15 items relating to depression. Internal consistency for the HSCL-25 has been reported to be 0.85 for the anxiety subscale and 0.91 for the depression subscale in a Norwegian sample [41].

Emotional distress was assessed using the SCL-5, based on the validated Norwegian adaption [2]. SCL-5 is comprised of five of the items from the HSCL-25: “Feeling fearful”, “Nervousness or shakiness”, “Feeling hopeless about the future”, “Feeling blue”, and “Worrying too much about things” [8]. Each item in both versions has four response options ranging from one (‘not at all’), to four (‘extremely’) and possible total scores on the SCL-5 range from 5 to 20. The SCL-5’s internal consistency has previously been reported to be between 0.77 and 0.87 [8, 17, 18]. The SCL-5 was not administered as a separate instrument; rather, the five items were extracted from responses in the HSCL-25 dataset.

Guided by a previous validation study [8], we calculated the mean score per item and, based on this score, adopted cut-offs for the presence/absence of emotional distress of 1.75 and 2.00 for the HSCL-25 and the SCL-5 respectively [8].

### Power calculation and statistical analyses

Given the reliance on a naturalistic and clinically derived sample, no a priori power analysis was conducted prior to data collection. Prior studies and methodological guidelines [42, 43] suggest that samples exceeding 100 participants are generally adequate to estimate Cronbach’s alpha. Also, for hypothesis testing, it is suggested that studies evaluating measurement properties of patient-reported outcome measures should preferably include at least 100 participants to obtain adequate estimates of validity and responsiveness [36–38].

The statistical analyses were performed using IBM Corp. SPSS, version 31.0.0.0. First, the item characteristics of the SCL-5 were examined by calculating missing values and floor/ceiling effects. According to the COSMIN guidelines, the proportion of missing data and the proportion of responses in the lowest and highest categories should be below 15% in order to provide robust reliability estimates and to enable change to be detected by an instrument [36].

ROC Curve analysis was performed to identify a cut-off point for the HSCL-25 and the SCL-5. With regard to the AUC it was hypothesised to be larger than 0.70 [38]. ROC is depicted as a curve which plots the sensitivity of the HSCL-25 and the SCL-5 against 1-specificity, using the two different cut-off points (1.75 and 2.0) recommended in previous research [8]. With regard to responsiveness, we hypothesised that at one-year follow-up the mean scores of the SCL-5 would significantly decrease compared to baseline. Mean scores were compared using an independent *t*-test [37].

### Ethical considerations

All patients and partners received verbal and written information about the study from staff not involved in the research. The original approval (No. 2012/683) was granted by the Regional Committee for Medical and Health Research Ethics in Central Norway. This approval was granted prior to data collection. An amendment related to extended psychometrical analyses was granted in 2022 (no.2012/683, 2022).

## Results

### Sample characteristics

A total of 174 participants were invited to participate in the study. Of these, 13 did not provide any SCL-5 baseline data. Hence, 161 participants (117 perpetrators and 44 partners) were included. The responsiveness sample consisted of a subset of this overall sample with 80 participants (58 perpetrators and 22 intimate partners). The socio-demographic characteristics of the overall sample are presented in Table 1.

**Table 1:** Baseline sample characteristics

Variables	Total sample (n = 161)			**Responsiveness sample**^**2**^ (n = 80)		
	**N**^**1**^	%	(SD)	**N**^**1**^	%	(SD)
**Age**						
Mean age (*SD*)	37.61		(10.14)	38.27		(10.39)
**Higher education**^3^	42	26.1		25	31.3	
**Mother tongue**						
Norwegian, %	138	85.7		69	86.3	
**Family situation**						
Living with intimate partner (%)	88	54.7		44	55.0	
**Employment**						
Unemployed (%)	28	17.4		14	17.5	
Full time work (%)	106	65.8		53	66.3	
Part time work (%)	24	14.9		11	13.8	

Note. ^1^N, denominator varies due to missing values; ^2^The number of perpetrators and victims who responded at the two time points; ^3^University level; *SD* = Standard deviation; *SCL-5, Symptom Checklist 5*

### Item characteristics and internal consistency

Missing responses were minimal; only three SCL-5 items (#2, #3 and #4) had any missing responses (1 value missing for each of these three items). An overview of the SCL-5 scores is shown in Table 2. The highest percentage with the lowest possible score (value 1) across the 5 items was 8.7%, indicating no floor effect assuming the 15% criterion [27]. Similarly, the highest percentage with the highest possible score (value 4) across the 5 items was 1.9%, indicating no ceiling effect [36]. Finally, Cronbach’s alpha for the SCL-5 total sample was 0.86, for perpetrators 0.83 and for partners 0.89, all indicating good internal consistency [38].

**Table 2:** Descriptive statistics for the SCL-5 items

	Total sample					**Responsiveness sample** ^**1**^		
Variables	Mean	(SD)	Kurtosis	Skewness	Missing	Mean	(SD)	Missing
1 Feeling nervous or shaky	1.65	(0.85)	0.29	1.12	0	1.54	(0.73)	0
2 Feeling fearful	2.25	(0.94)	−0.81	0.27	1	2.13	(0.97)	0
3 Feeling hopeless about the future	2.14	(0.90)	−0.60	0.40	1	2.11	(0.88)	1
4 Feeling blue	2.03	(0.85)	−0.21	0.56	1	2	(0.9)	0
5 Worrying too much	2.18	(0.91)	−0.43	0.53	0	2.15	(0.94)	0

Abbreviations. ^1^The number of perpetrators and victims who responded at baseline and one year follow-up; *SD* = Standard deviation; *SCL-5, Symptom Checklist 5*

#### Perpetrators

Overall, 112 of the perpetrators completed all HSCL-25 items at the first assessment. Three completed 24 of the items, and two completed 23 of the items. These ratings were imputed for the analyses, with missing items replaced by the mean total score of the rating (i.e. all 117 of the perpetrators were included in the analyses). Of these 117 cases, 53 (45.3%) were identified as clinical cases using the HSCL-25 cut-off. In ROC-analysis testing the SCL-5 capability to identify clinical cases, the area under the curve was 93.4% (95% CI: 0.891-0.976).

#### Victims

Forty-three partners answered all HSCL-25 items at the first assessment. One partner completed only 24 of the items. This rating was imputed for the analyses by the same method as used above for the perpetrators (i.e. 44 of the partners were included in the analyses). Of these 44 cases, 25 (56.8%) were identified as clinical cases using the HSCL-25 cut-off. In ROC-analysis testing the SCL-5 capability to identify clinical cases, the area under the curve was 96.9% (95% CI: 0.922–1.017).

#### Total sample, perpetrators and victims combined

**Fig. 1:**
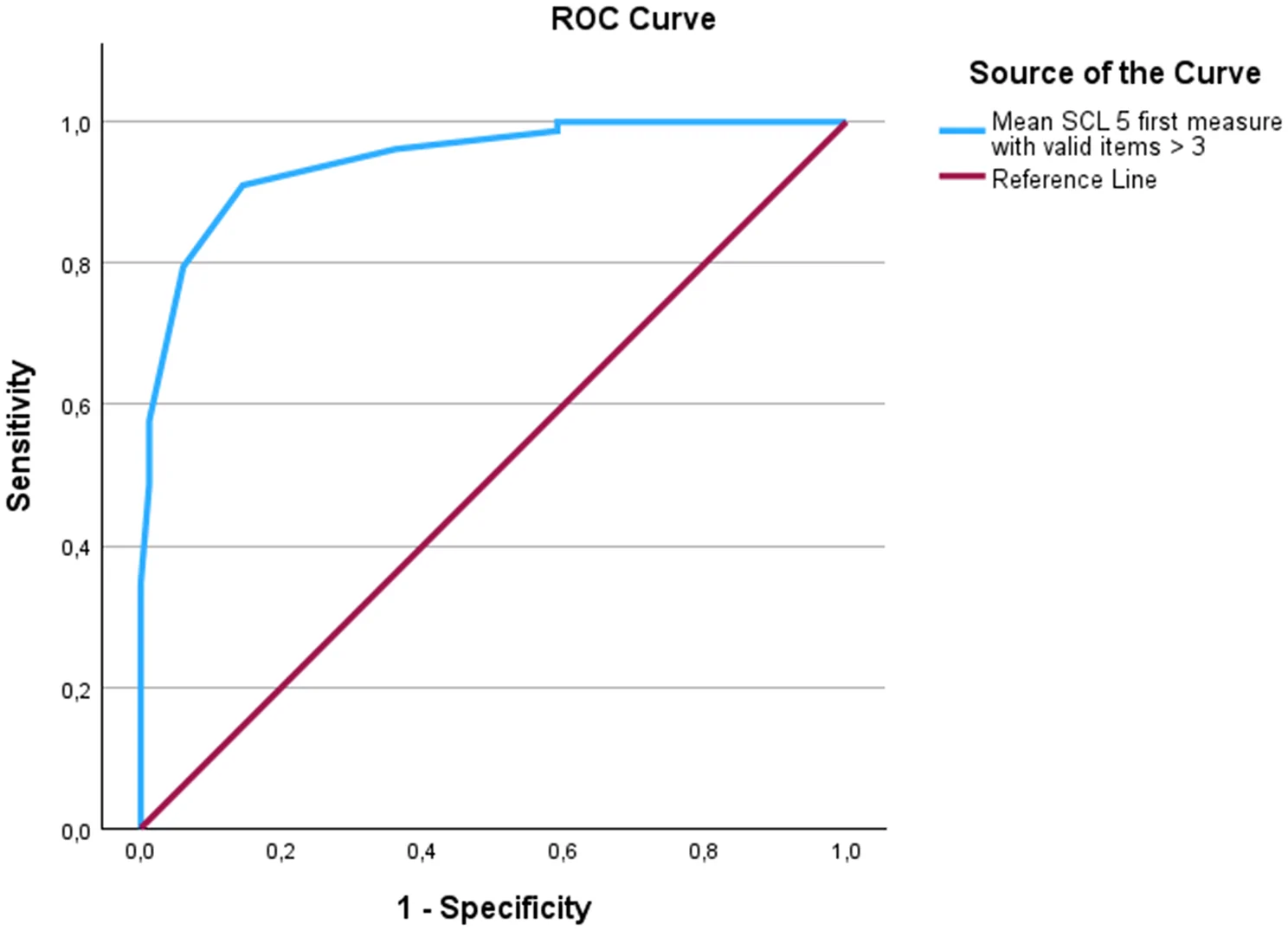
Sensitivity and specificity of SCL-5 compared with HSCL-25 total sample

For ROC-analysis of the HSCL-25 and the SCL-5 the scores from the baseline rating of the HSCL-25 were used. Of these 161 cases, 78 (48.4%) were identified as clinical cases using the HSCL-25 cut-off specified above. In ROC-analysis testing the SCL-5 capability to identify clinical cases, area under the curve was 94.6% (95% CI: 0.913-0.978), indicating that the SCL-5 has good psychometric properties. The ROC curve of the total sample of perpetrators and victims combined is depicted in Fig. 1.

### Responsiveness to change

Regarding responsiveness, we set a criterion of statistical significance (*p* < 0.05) in the reduction in mean scores from the baseline to the one-year follow-up as a cut-off for judging adequate responsiveness of the SCL-5. The sample mean score fell from 1.98 to 1.74 and this mean change was indeed statistically significant (*t* −4.021, CI −0.360 to −0.121; *p* < 0.001). The mean total score on the participants’ SCL-5 decreased in 54% of cases *(n* = 43).

Overall, in relation to the COSMIN guidelines, the proportion of correctly predicted hypotheses was 100% (4/4), indicating that the SCL-5 has good psychometric properties [38].

## Discussion

### Main findings

Our study aimed to investigate the psychometric properties of the SCL-5 amongst perpetrators and victims of IPV. Regarding the quality of data, we observed very few missing values, and no floor or ceiling effects were found in our analyses. According to the COSMIN guidelines [36], the proportion of missing data and the proportion of responses in the lowest and highest categories should be below 15% in order to provide robust estimates and to detect change within an instrument. The very low proportion of missing values here is well within this requirement and indicates that the short version is not burdensome to participants. Similarly, the small proportion of highest (<9%) and lowest (<2%) response category scores implies that almost all of the full range of possible responses are captured by the short instrument, and it is unlikely that a large number of outliers are being excluded from assessment.

Concerning internal consistency, Cronbach’s alpha exceeded 0.70 for the total sample, which is in line with the predefined hypothesis value, and is considered good [38]. Our result aligns with that in another study validating the SCL-5 in a general population, where the Cronbach’s alpha was > 0.8 [8], and is comparable to the results of a Spanish validation study on primary care patients, which had an α value of 0.77 [17]. Taken together, these studies and our own provide evidence of internal reliability in various populations which is at least adequate for its use as a screening tool.

In terms of the calculation of the area under the ROC curve, values can range from 0.5 to 1.0 [38]. In line with a previous study [17], we hypothesised the AUC to be larger than 0.70 [38]. Using the HSCL-25 as the reference measure, the shortened version, SCL 5, demonstrated high accuracy in case-detection here. With the previously proposed 2-point cut-off defining a clinical case [8], 89% of full version clinical cases were identified by the short version. This finding is similar to other studies analysing the validity of the SCL-5 in other populations [8, 17, 18]. From a clinical perspective, the successful classification of cases suggests that the SCL-5 represents a viable alternative screening tool for clinicians and mental healthcare professionals when attempting to identify anxiety and depression symptoms in IPV-related clinical settings. It reduces the respondent burden and thus potentially will improve completion rates [13].

It is important to note for this analysis in particular that the SCL-5 was not administered as a separate questionnaire in the present study. Instead, the five items comprising the SCL-5 were extracted from patients’ responses to the full HSCL-25 version. This approach was particularly appropriate in our busy clinic, as it allowed us to reduce the number of self-rated scales that participants needed to complete. Similar methodological approaches have been used in previous validation studies conducted in Germany [16], Spain [17], and Norway [8]. Nevertheless, this extraction of items from the HSCL-25 represents a limitation, as it is conceivable that responses may differ if the scales are administered independently.

The mean score on the SCL-5 decreased substantially from 1.98 at baseline to 1.74 (12.1%) at the one-year follow-up and this change was statistically significant. This indicates that the intervention here which primarily targeted the emotion of anger was also associated with overall reductions in anxiety and depression as well. This in turn highlights the need for awareness of the extensive overlap between these emotions in this population [30] and the potential they have to mutually reinforce each other before, during and after stressful encounters. Nearly half (46%) of the perpetrators here (and over half of the victims) had clinical levels of anxiety and/or depression based on the full HSCL-25 criteria for caseness. In relation to the study objectives the substantial reduction here also indicates that the SCL-5 is sensitive enough to detect change over time in this population and thus is suitable for therapeutic evaluations. As a growing body of research has indicated a clear link between IPV and anxiety and depression amongst both victims and perpetrators, this study suggests that symptoms of anxiety and depression should be routinely measured in both groups when they come into contact with services. Our findings are clinically relevant and warrant using the SCL-5 to identify people that might need psychological treatment for anxiety and depression alongside their IPV issues.

Nevertheless, it should be noted that the mean score decreased for only 54% of participants after one year. This means that almost half (46%) of the sample did not change in a positive direction. This absence of change can have several explanations. Firstly, the anger management treatment was a manualised therapy specifically targeting angry thoughts and feelings and violent behaviour, without explicitly aiming for change in feelings of anxiety and depression. We have just noted the mutual interplay of anger, anxiety and depression in the perpetrator population but inevitably changes in the latter emotions could be viewed only as a side-effect, albeit positive, of the group therapy. Such an indirect effect may be weaker than any impact on the primary emotion of anger which is directly targeted by the therapy. Secondly, the responsiveness sample consisted of both perpetrators and partners, and only the perpetrators received therapy. The rationale for combining the two groups in this analysis was the possibility that any improvements in the mental health of the perpetrators could lead to improvements amongst partners if the couple are still in contact as another potential side-effect of the treatment. Even so, this finding underscores the significance of providing support and treatment for IPV perpetrators, providing integrated care which addresses their psychological distress alongside their violent behaviour. Our findings, in conjunction with previous research [21], highlight the need for comprehensive and multidisciplinary interventions which address the victim partners’ mental health concerns [44].

Whilst the study demonstrated a decrease in SCL-5 scores, further research is necessary to explore the robustness of the SCL-5 in detecting symptoms of anxiety and depression in a population of perpetrators of IPV over longer time periods [45]. As stated in a recent systematic review [46], there is still a clear gap in knowledge regarding the long-term impact of violence treatment on the mental health outcomes of perpetrators of IPV.

### Strengths and limitations

This study is the first, to our knowledge, examining the psychometric properties of the SCL-5 in a sample of victims and perpetrators of intimate partner violence. Thus, the application of the SCL-5 amongst such perpetrators and their partners is a new context to consider when evaluating interventions aimed at improving the mental health of these populations. Nonetheless, several factors should be considered which may impact the generalisability of the results. The study sample consisted entirely of male perpetrators of violence towards a female partner which limits the generalisability of the findings to other populations (e.g. same sex relationships). This study was originally part of a clinical trial [40] and follow up scores were by definition potentially affected by exposure to the active and comparator interventions. In addition, it has been suggested that men voluntarily attending treatment are more educated, more often share a household with their partner, and more often report problems with anxiety and depression, than those mandated to treatment [47]. Hence, our study population may differ from those mandated by the courts to undergo treatment. However, as IPV occurs within the family sphere, a large group of perpetrators are not reported to the police or convicted. Thus, from a public health perspective, the population in this study represents the broader population of individuals involved in IPV.

Given the naturalistic and clinically derived sample in this study, no a priori power analysis was conducted prior to data collection. However, prior studies and methodological guidelines [36–38, 43] suggest that samples exceeding 100 participants are very good to estimate Cronbach’s alpha, so the overall sample size was deemed appropriate for estimating reliability coefficients for the five-item scale [42].

Subgroup analyses conducted separately for perpetrators and partners were exploratory in nature as they were inevitably based on smaller sample sizes, especially for the sample of partners (*n* = 44). These analyses should therefore be interpreted with caution, as statistical power within subgroups was limited, particularly for detecting a small effect size. However, participants were recruited from a consecutive series of perpetrators presenting at a clinic with very few refusals and thus those eligible to participate clearly represent the population of those voluntarily seeking help for IPV in this setting. Nevertheless, the participant selection process could have introduced unknown biases, as certain groups of eligible participants could have systematically avoided participation due, for instance, to lack of motivation to change.

The study relied entirely on self-reporting, which is always subject to individual interpretation and potential response biases. This is especially important to consider within the context of domestic violence, as violence in the family is still a hidden phenomenon where those involved may underreport mental health problems or, conversely, may over-report them in order to mitigate anticipated punishment by the criminal justice system [20]. Finally, it is a limitation that this study used the HSCL-25 as a reference measure. Even though the HSCL-25 is a validated screening questionnaire to measure anxiety and depression [2], it is not a diagnostic gold standard. Furthermore, we did not investigate construct validity through associations with theoretically related constructs beyond the HSCL-25. Instead, the five items comprising the SCL-5 were extracted from patients’ responses to the full HSCL-25 version, limiting our findings regarding the construct validity of the SCL-5. Consequently, these limitations have implications for future research. Ideally, future psychometric studies should administer the SCL-5 with theoretically related constructs and with a true gold standard clinical interview to further evaluate the construct validity in IPV-related clinical settings. Additionally, future studies should examine associations between SCL-5 scores and related psychological constructs to enable a more comprehensive investigation of construct validity. Furthermore, considering these limitations, further research with larger, more diverse, clinical populations will enhance the generalisability of future findings in this field.

## Conclusion

The SCL-5 showed adequate psychometric properties, even though it requires less time and resources to complete compared to the longer instrument from which it is derived. Hence, based on our findings, it can be considered a convenient yet robust tool to detect symptoms of anxiety and depression in a population of men voluntarily seeking treatment for IPV and their victim partners.

## Data Availability

The data are not available due to ethical reasons.

## Abbreviations

AUC: Area Under the Curve
CBGT: Cognitive behavioural group therapy
CI: Confidence Interval
GT: Group therapy
HSCL-25: Hopkins Symptom Checklist 25 items
IPV: Intimate Partner Violence
MBSR: Mindfulness-based stress reduction
ROC: Receiver Operating Characteristics
SCL-5: Symptom Checklist-5 items
SD: Standard deviation
